# Assessment of direction changes in waste electrical and electronic equipment management in Poland

**DOI:** 10.1007/s11356-024-34227-4

**Published:** 2024-07-06

**Authors:** Grzegorz Przydatek, Włodzimierz Kanownik

**Affiliations:** 1 Engineering Institute of University of Applied Sciences in Nowy Sącz, Zamenhofa 1a, Nowy Sącz, Poland; 2 Department of Environmental Engineering and Geodesy of the Agricultural University of Krakow, Al. Mickiewicza, 24-28 Krakow, Poland

**Keywords:** Collection, Recovery, Recycling, Waste management, e-waste, Statistics

## Abstract

A growing amount of waste electrical and electronic equipment (WEEE) indicates the need to verify the effectiveness of its management both nationally and globally. An analysis of the WEEE economy in Poland conducted over 5 years confirmed a 16.64% increase in the mass of collected equipment. The maximum annual mass of electrical and electronic equipment introduced to the market during this period was 607,240 Mg, with the average value exceeding 500,000 Mg. The WEEE category with the largest collected mass was waste code 20 01 36, which exceeded 235,000 Mg, whilst the highest waste weight accumulation rate of 45.98 kg per capita was recorded in one of the smallest voivodeships in Poland. This result showed the diversity of WEEE accumulation on a national scale. Overall, a noticeable increase in the WEEE accumulation rate has occurred as Poland’s gross domestic product has increased, despite a decreasing population. An analysis based on the waste accumulation indicators, including socioeconomic factors, confirmed the need to develop forms of WEEE recovery and recycling to transition to a circular economy and promote the synergy of activities amongst all players in WEEE management.

## Introduction

Electronic waste, or waste electrical and electronic equipment (WEEE), comprises used equipment that has reached the end of its economic life and can no longer be used by consumers (Işıldara et al. 2019). This often consists of e-waste, which includes consumer lighting equipment, electrical and electronic tools, toys, leisure and sports equipment, medical devices, monitoring and control instruments, and automatic dispensers (Patil and Ramakrishna 2020).

Globally, the end-of-life processing of WEEE includes reuse, repair, refurbishment, repurposing parts into other products, recycling, resource recovery, landfill (both controlled disposal and compliant disposal), incineration, and bulk landfill (Maes and Preston-Whyte 2022). Notably, WEEE is currently one of the largest waste streams in the world by volume (Chung et al. 2011) since electrical and electronic equipment is being purchased by an increasing number of individuals and institutions. Additionally, WEEE includes hazardous waste, which results from items containing lead, mercury, chromium, cadmium, and lithium, amongst other elements (UNEP 2020). According to Zuo et al. (2020), the improper disposal of e-waste can threaten human health and the environment due to the toxic substances it often contains. Therefore, the handling and disposal of such waste require special attention to avoid the risk of environmental pollution and danger to humans (Araujo et al. 2017).

According to European Union (EU) Directive 2012/19/EU (EU 2012), also known as the ‘WEE Directive’, WEEE includes the following equipment: information technology and telecommunications equipment, consumer equipment and photovoltaic panels, lighting equipment, electric power tools, toys and recreational and sports equipment, medical equipment, monitoring and control instruments, and vending machines (Araujo et al. 2017). The WEEE Directive also introduces requirements for the processing of various e-waste materials and components as well as the location of waste storage sites whilst maintaining environmentally sustainable recycling strategies. Parajuly and Wenzel (2017) argued that the e-waste stream has a high potential for the reuse and recovery of valuable materials.

The handling and disposal of such hazardous waste require special attention to avoid environmental pollution. In this context, the hierarchy of waste management, which is determined by Directive 2008/98/EC (EC 2008), remains essential. As a result, EU Member States take measures to encourage solutions that minimise the amount of waste generated (Gharfalkar et al. 2015).

Andeobu et al. (2021) identified the main problem in the WEEE economy as the lack of effective collection and recycling systems and mechanisms to hold electrical and electronic equipment (EEE) producers accountable for the disposal of end-of-life equipment. Przydatek (2019) showed differences in selected aspects of waste management in the continental dimension, which resulted from the lack of legal regulations and infrastructure facilities in this field. Patil and Ramakrishna (2020) reported that shortcomings in waste management are due to insufficient financial resources for e-waste recycling. Therefore, the environmental and health consequences of improper e-waste disposal have attracted the attention of consultative non-governmental organisations (NGOs), including the World Health Organization (WHO), Basel Action Network, and United Nations Environment Programme (UNEP) (Schumacher and Agbemabiese 2019; Murthy and Ramakrishna 2022).

According to the provisions of the Act of September 11, 2015, on WEEE in Poland (Act 2015), waste collectors, the operators of treatment plants, the operators of recycling installations and recovery processes other than recycling, and end users are required to limit the amount of WEEE and reuse, recycle, and apply other forms of recovery for the more efficient use of resources and secondary raw materials.

Challenges in the e-waste economy include illegal storage and export, as well as issues related to the consumer cost of e-waste recycling (Lee and Na 2010). Bhaskar and Turaga (2018) noted that in the traditional model, waste management is usually financed by taxpayers, although some countries have introduced a fee for waste producers. This financing scheme has proven helpful in treating WEEE in developed countries. For example, as part of its WEEE disposal activities, the Japanese government has established a financing system involving producers and consumers (Zhang et al. 2020).

Dynamic progress and changes in EEE technology have resulted in the faster ageing of functioning products, thereby increasing the amount of e-waste, which indicates the need to develop new methods of WEEE recycling and disposal (Kumar and Rawat 2018). Therefore, innovations and improvements are necessary through new and efficient products and services, in line with a globally significant closed economy (Xavier et al. 2021).

This article aims to assess the direction of changes in the WEEE economy in Poland during the 2014–2018 period whilst considering WEEE accumulation indicators—including socioeconomic factors—in the context of developing their recovery and recycling processes.

## WEEE management in Poland

Poland is located in Central Europe and has an area of 322,575 km^2^. In terms of administration, a voivodeship is the largest unit of the basic territorial division of the country. The Masovian Voivodeship (central Poland) makes up the largest area of 35,579 km^2^, whilst the smallest area of 9412 km^2^ falls under the Opole Voivodeship (southwestern Poland) (Przydatek 2020).

The management system for WEEE and used batteries and accumulators begins with the individual or institutional consumer deciding to dispose of these products (constituting waste for the consumer at this point). The consumer has two options for this waste. First, WEEE can be handed over to municipal selective waste collection points. Second, returning a used product is often possible since the entities introducing EEE to the market are obliged to collect similar equipment from the consumer. In many cases, processing plants can dismantle these products to recover the main raw materials. As a result, valuable materials are obtained (e.g. steel, non-ferrous metals, and plastics), which can be transported to appropriate recycling plants (Brooks et al. 2019). In the case of WEEE from households delivered to collection points, EEE producers are responsible for the costs of its collection, treatment, recovery, and disposal at a minimum.

Banaszkiewicz et al. (2022) showed that collection activities in Poland do not keep up with the increase in the amount of EEE in the market. Moreover, the increased volume of heavy long-life equipment introduced may cause problems with achieving collection goals, whilst approximately 5% is exported from Poland as WEEE (Puckett et al. 2018).

## Materials and methods

For the analysis, annual qualitative and quantitative data for the 2014–2018 period were obtained based on surveys addressed to marshal offices in 16 Polish voivodeships and the annual reports of the Chief Inspectorate of Environmental Protection (IEP) (the central body of the Polish state administration responsible for collecting, sharing, and disseminating statistical data). The analysed data covered the mass of waste collected in the form of WEEE and broken down into five types by code (Regulation 2020):

- 20 01 21*—fluorescent lamps and other wastes containing mercury
- 20 01 33*—batteries and accumulators (lead batteries and accumulators, nickel–cadmium batteries and accumulators, mercury-containing batteries) and unsorted parts containing these batteries
- 20 01 34—batteries and accumulators other than those mentioned in 20 01 33
- 20 01 35*—WEEE other than those mentioned in 20 01 21 and 20 01 23 containing hazardous components
- 20 01 36—WEEE other than those mentioned in 20 01 21* and 20 01 35*
  *Hazardous waste in the waste catalogue is waste marked with an upper index in the form of an asterisk ‘*’ next to the waste type code, apart from the provisions of Art. 7 of the Act of 14 December 2012, on waste (Przydatek et al. 2022).

The analysis also used data from the IEP regarding the mass of EEE generated in Poland and the number of companies operating in the field of waste collection in the register each year (Report 2014). Data from Statistics Poland (SP) provided information on the mass of processed EEE in the country, the number of inhabitants in individual voivodships, the annual gross domestic product (GDP), and the annual disposable income per capita (DIC) (i.e. the sum of current household income from all sources minus advances for income tax, taxes, and social and health insurance contributions) (SP 2014–2018).

Based on the aforementioned data, the percentage shares of the five types of waste collected in Poland during the 2014–2018 period were calculated. For each year, the level of WEEE processing was determined as a percentage of the mass of waste equipment collected to the mass of processed equipment, whilst the level of waste equipment collection was calculated as the mass percentage of collected waste equipment to the equipment introduced in the previous year. Annual WEEE waste accumulation rates were reported in kilograms per inhabitant of Poland in individual (16) voivodeships. Economic ratios were calculated as the annual mass of WEEE collected to the annual GDP in billions of USD and DIC in PLN (the monetary unit in Poland). Some researchers have also used waste accumulation and socioeconomic indicators (Li et al. 2011; Zorpas et al. 2017; Leclerc and Badami 2020). Przydatek and Ciągło (2020) showed that many indicators of waste accumulation can help identify the direction of changes in waste management, considering local geographical conditions, socioeconomic factors, and legal regulations.

The statistical analysis included the maximum, minimum, and average of these measures. The correlation between the annual mass of WEEE collected per inhabitant of Poland and the mass of equipment introduced in the previous year, the annual GDP, and the annual DIC were checked. Additionally, Pearson’s linear correlation coefficient *r* and coefficient of determination *R*^2^ (*p* < 0.05) were calculated (Przydatek and Kanownik 2019), with Statistica 12 (StatSoft Poland, StatSoft, Inc., USA) being used for statistical analysis.

## Results

The mass of WEEE collected during the 2014–2018 period, broken down by 16 voivodeships, is presented in Table 1. The largest mass of WEEE collected during this period ranged from 41 694.82 to 82 799.21 Mg, indicating an increase in the largest area, the Masovian Voivodeship (No. 8). On the other hand, the lowest mass of collected equipment ranged from 841.72 to 606.60 Mg, a clear decrease in the smallest area of the Opole Voivodeship (No. 12). Notably, the total values in the two voivodeships differed significantly by more than 300,000 Mg. On a national scale, an increase in collected WEEE was observed, with a total collect mass of 1,047,618 Mg.

**Table 1:** Mass of WEEE collected in individual Polish voivodeships during the 2014–2018 period

Voivodeship	No	2014	2015	2016	2017	2018	2014–2018
				(Mg)			
West Pomeranian	1	849.90	1271.40	1316.35	1556.97	1557.18	6552
Pomeranian	2	4668.87	1713.83	3676.78	3130.55	2738.63	15,929
Warmian-Masurian	3	1065.46	1274.40	1369.61	1535.27	1603.52	6848
Podlaskie	4	1606.78	1332.74	2598.30	3805.07	1514.66	10,858
Lubuskie	5	4462.53	12,348.89	2641.11	2460.43	2245.19	24,158
Greater Poland	6	13,461.97	3592.87	3513.72	10,482.52	12,597.27	43,648
Kuyavian-Pomeranian	7	1504.84	2362.53	7506.66	13,351.60	21,144.43	45,870
Masovian	8	41,694.82	55,254.68	73,673.67	81,435.47	82,799.21	334,858
Lower Silesia	9	6403.88	5831.35	9215.28	12,236.90	11,614.58	45,302
Lodz	10	21,016.14	18,820.19	13,867.26	23,391.43	33,361.88	110,457
Lublin	11	7537.72	12,099.17	22,526.53	20,535.40	30,311.60	93,010
Opole	12	841.72	554.02	811.91	712.63	606.60	3527
Silesian	13	6731.77	7177.12	10,140.84	9675.29	6729.16	40,454
Świętokrzyskie	14	19,472.17	23,628.54	41,931.74	43,034.49	57,078.69	185,146
Lesser Poland	15	15,227.84	8964.63	11,034.01	14,198.91	11,568.50	60,994
Subcarpathian	16	2181.41	2435.92	3249.69	6212.06	5927.81	20,007
Total		148,728	158,662	209,073	247,755	283,399	1,047,618

The largest mass of WEEE collected based on type was code 20 01 36 (i.e. used electrical and electronic devices other than those mentioned in 20 01 21 and 20 01 35* containing hazardous components). This increase in the amount of WEEE collected since 1 January 2016 stems from the fact that the entities introducing equipment (not only equipment intended for households as before) are obliged to achieve certain minimum annual levels of waste equipment collection for code 20 01 36 items (Koc-Jurczyk and Jurczyk 2017). Over the 5-year study period, the collected mass of waste code 20 01 36 more than doubled, from 112,264.87 to 235,508.52 Mg (i.e. by over 123,000 Mg), with a total value of 844.624 Mg (Table 2). The smallest mass of collected equipment was observed for the hazardous waste code 20 01 21* (fluorescent lamps and other waste containing mercury), which was 8398.76 Mg for the 2014–2018 period. Notably, this waste is characterised by the highest growth, from 808.49 Mg in 2014 to 2398.25 Mg in 2016 (i.e. nearly triple).

**Table 2:** Mass of WEEE collected in Poland during the 2014–2018 period, by code

Code of waste	2014	2015	2016	2017	2018	2014–2018
			(Mg)			
20 01 21*	808.49	808.49	2398.25	2274.34	2116.72	8398.76
20 01 33*	2639.13	2848.56	3578.02	2338.27	3874.70	15,278.68
20 01 34	2759.55	1741.09	2634.41	3941.91	3795.70	14,872.66
20 01 35*	30,256.18	34,132.79	32,256.91	29,694.61	38,103.28	164,443.76
20 01 36	112,264.87	119,138.88	168,205.87	209,505.86	235,508.52	844,624.00

*Hazardous waste in the waste catalogue is waste marked with an upper index in the form of an asterisk

**Fig. 1:**
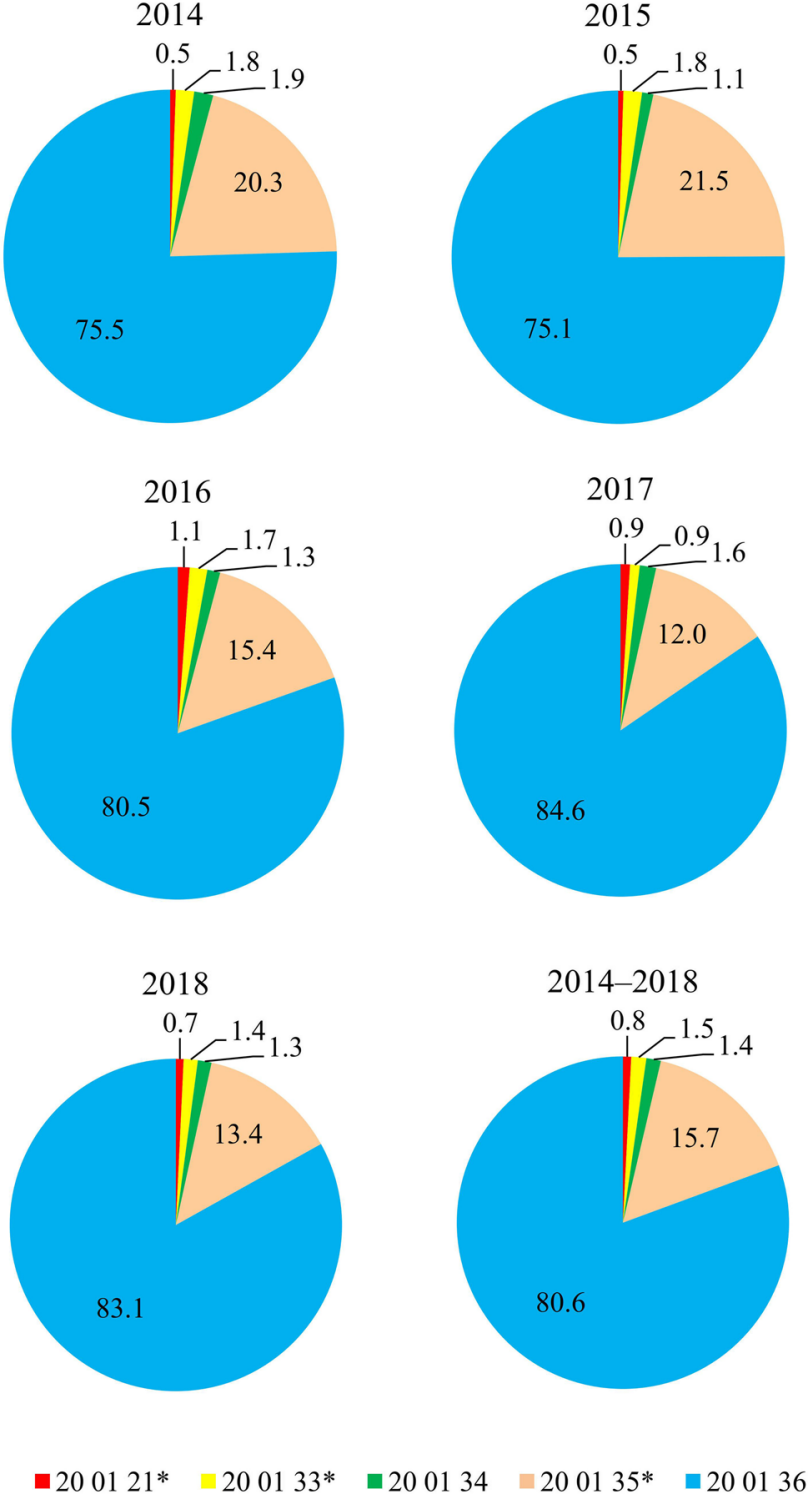
Mass percentage by waste type (WEEE code number) collected in Poland during the 2014–2018 period

In the analysed years, the largest share (exceeding 70%) was constituted by WEEE with the code 20 01 36. WEEE containing hazardous components other than those listed in 20 01 21 (i.e. 20 01 35*) had the second highest share, with 21.5% in 2015—reduced to 12.0% in 2017. On the other hand, the lowest share (not exceeding 1%) comprised hazardous fluorescent lamps and other waste containing mercury (20 01 21*), as shown in Fig. 1. The share of the remaining analysed waste did not exceed 2% of the total mass of WEEE collected in a given year.

The highest mass of collected equipment (283,399 Mg) was observed in 2018, whilst the highest mass of processed WEEE (256,035 Mg) was observed in 2017; these figures showed noticeable increases of over 130,000 and 90,000 Mg, respectively. The average mass of collected WEEE was 6721 Mg higher than that of processed equipment. Significantly higher values were recorded for the mass of EEE introduced to the market, as confirmed by the average result exceeding 500,000 Mg. A significant increase of over 120,000 Mg in introduced equipment yielded a range of 486,180–607,240 Mg, with the highest increase being observed in 2018. Additionally, the number of entities operating in waste equipment collection increased, exceeding 1000 by 2018.

Despite a 0.11% decrease in the population of Poland, the share of WEEE processed in the five analysed years increased by 5.2%, whilst that of WEEE collected rose by 16.64%. The average percentages of the listed items differed significantly, with the predominance being attributable to the processed equipment (over 50%). The highest WEEE weight accumulation index was 7.46 kg per capita in 2018. This parameter was also characterised by an annual increase of 3.55 kg per capita.

GDP represents an important indicator at the national scale and has increased in Poland by as much as USD 44.9 billion. In turn, the annual DIC increased by PLN 4236. Similarly, the indicators covering the mass of WEEE collected per unit GDP and the mass of WEEE collected per disposable income increased by 208 Mg billion USD^−1^ and 4.7 Mg PLN^−1^, respectively (Table 3).

**Table 3:** Basic indicator data on the WEEE economy in Poland during the 2014–2018 period

Parameter	2014	2015	2016	2017	2018	Average
Mass of collected WEEE (Mg)	148,725	158,662	209,073	247,755	283,399	209,523
WEEE processed (Mg) (SP)	162,363	168,942	223,867	256,035	-	202,802
Mass of introduced EEE (Mg) in the previous year	486,180	518,868	526,914	583,147	607,240	542,976
Number of enterprises operating in the field of collecting waste equipment	1059	898	2228	2253	-	1610
Polish population (thousand)	38,010	37,990	37,970	37,970	37,970	37,982
GDP (billion USD)	542.5	477.8	472.6	526.5	587.4	521
Annual disposable income per person (PLN)	16,080	16,632	17,700	19,176	20,316	17,981
WEEE processing level (%)	91.6	93.9	93.4	96.8	-	94
WEEE collection level (%)	30.6	30.6	39.7	42.5	46.7	38
Weight (kg) of collected WEEE per cap	3.91	4.18	5.51	6.53	7.46	6
Mass of collected WEEE per unit of GDP (Mg bilion USD^−1^)	274	332	442	471	482	400
Mass of WEEE collected per disposable income per person (Mg PLN^−1^)	9.2	9.5	11.8	12.9	13.9	11

**Fig. 2:**
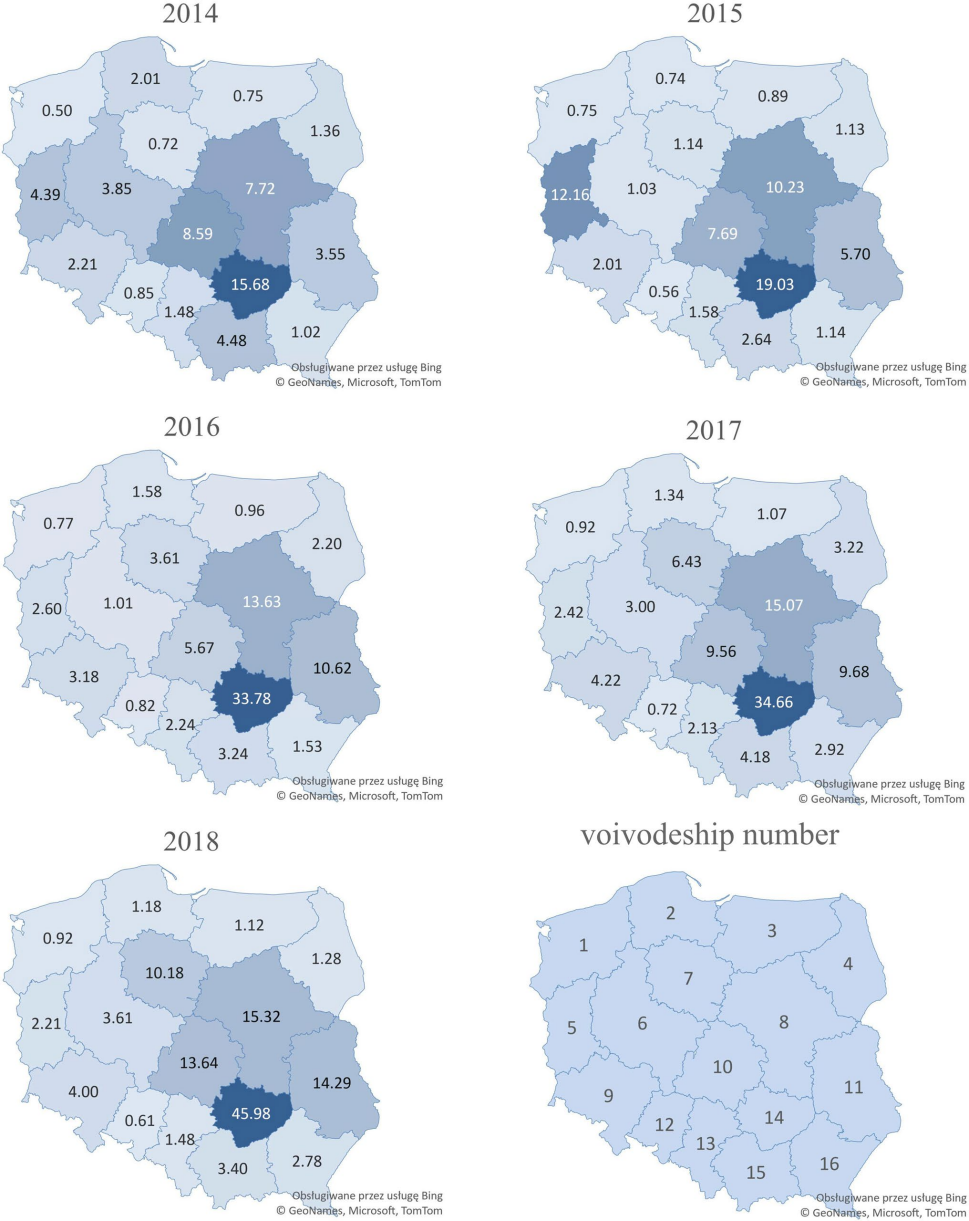
Mass (kg) of collected WEEE per capita in Polish voivodeships during the 2014–2018 period

Figure 2 presents the WEEE accumulation rates per capita in individual voivodeships during the 2014–2018 period. The dominance in the Świętokrzyskie Voivodeship (No. 14) is notable, showing a noticeable increase of 15.68–45.98 kg per capita per year. In other voivodeships, this indicator did not exceed 15 kg per capita—even in the Masovian Voivodeship (No. 8). On the other hand, the lowest WEEE accumulation rates of 0.50 and 0.61 kg per capita per year were recorded in West Pomeranian (No. 1) in 2014 and Opole (No. 12) Voivodeship in 2018, respectively. The latter experienced a clear decrease.

**Fig. 3:**
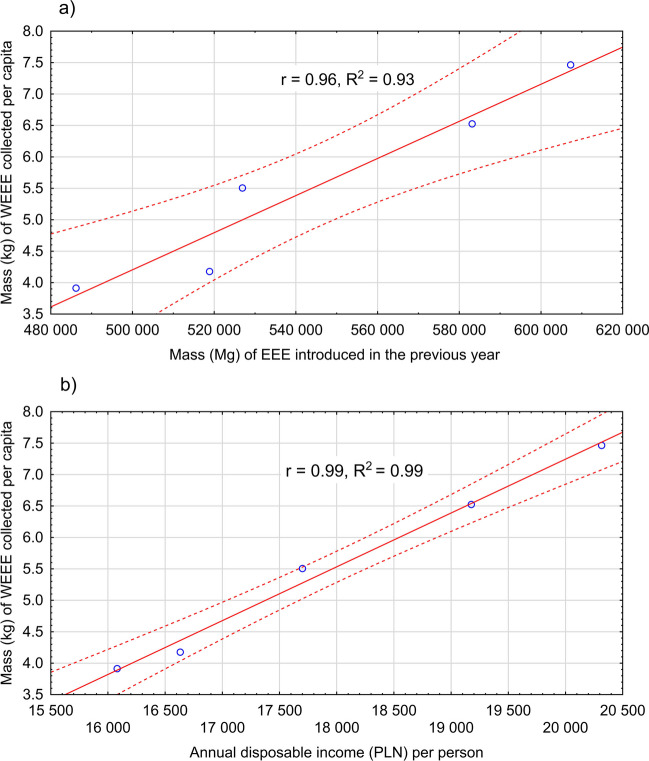
**a** Relationship between the mass of EEE introduced in the previous year. **b** Annual disposable income (PLN) per person and the mass (kg) of WEEE collected per capita

The statistical analysis showed a statistically significant correlation (*r* = 0.96) between the WEEE accumulation rate per capita and the mass of EEE introduced in the previous year (Fig. 3a). A similarly significantly static relationship occurred between the annual disposable income per capita and the WEEE accumulation index per capita (*r* = 0.99), with a high determination index (*R*^2^ = 0.99) (Fig. 3b).

## Discussion

The amount of generated e-waste is growing rapidly, which indicates the need to develop recycling technologies. Additionally, Perkins et al. (2014) noted a significant 18-fold increase in generated e-waste in India.

Parajuly et al. (2020) predicted that the rate of waste generation would double. According to Adamcová and Vaverková (2016), this is a consequence of the Industrial Revolution. Similarly, in Poland, a noticeable increase of over 130,000 Mg in the mass of collected WEEE was noted during the 2014–2018 period, corresponding to the global trend (Cui and Anderson 2016). The progressive increase in EEE consumption is associated with the problem of its rational management, which requires new solutions since the composition of electronic devices is rapidly changing with technological progress (Arduin et al. 2020).

The largest mass of collected WEEE amounted to 82,799.21 Mg in 2018 in the Central Polish Voivodeship, which is distinguished by having the largest area and population (Przydatek et al. 2021). The number of EEE introduced to the market was higher in this area, which is confirmed by the high mass of 607,240 Mg being recorded in 2018, with the average exceeding 500,000 Mg.

The largest mass of WEEE collected by type was for 20 01 36 (i.e. WEEE other than those listed in 20 01 21*, 20 01 23*, and 20 01 35*), amounting to 235,508.52 Mg in 2018—an increase of over 123,000 Mg over the studied period. In this context, despite a noticeable decrease in the population of Poland over the last 5 years, the share of WEEE collected and treated increased by 16.64 and 5.2%, respectively. Shittu et al. (2021) forecasted an increase in WEEE generation of 3–5% per year. The highest WEEE accumulation index in Poland was 7.46 kg per capita in 2018. In contrast, a significantly higher WEEE accumulation result of 45.98 kg per capita was recorded in one of the smallest voivodeships in Poland. In turn, Przydatek and Ciągło (2020) highlighted a higher indicator value of more than 21 kg in one of the communes of southern Poland, indicating the diversity of results on a national scale. Furthermore, Diedler et al. (2018) reported that in Germany and Serbia, the rates were higher and amounted to 22.8 and 11 kg per capita per year, respectively. This suggests that the accumulation of waste in the environment should receive greater social awareness due to the problems caused by its increase (Matsakas et al. 2017).

**Fig. 4:**
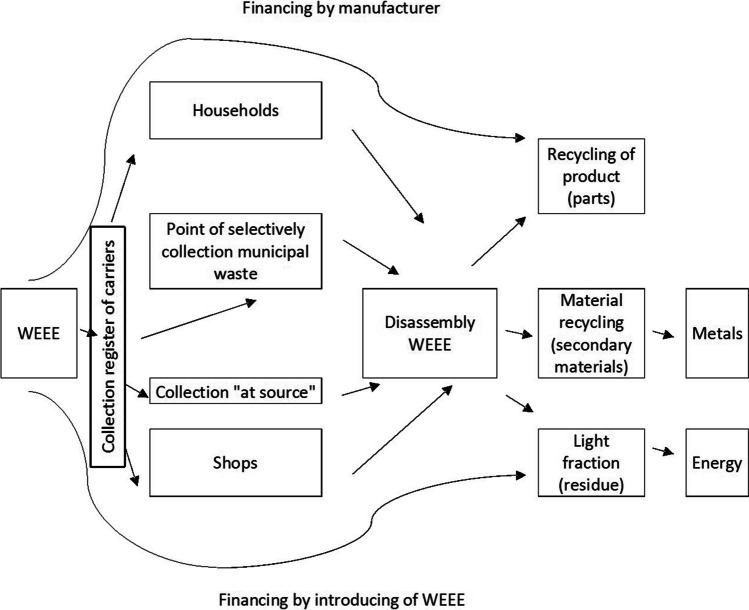
Model of financing WEEE management

In Poland, WEEE generated in households is collected at selective municipal waste collection points and shops. This process is financed by EEE producers, which includes export, recovery, and disposal (Fig. 4).

Some researchers (Mota et al. 2015; Zsigraiová et al. 2013) have emphasised that optimising the number of vehicles and containers can affect collection costs and environmental benefits due to lower emissions. Moreover, Ismail and Hanafiah (2017) recognised WEEE as valuable secondary raw materials, whose effective management is advisable through recycling.

Along with technological progress, the amount of e-waste and associated management costs increase (Oteng-Ababio 2010). This aligns with the increase in the mass of WEEE collected per unit GDP and the mass of WEEE collected per unit of disposable income in Poland, which amounted to 208 Mg billion USD^−1^ and 4.7 Mg PLN^−1^, respectively. Other researchers (Sousa et al. 2018) have also noted an increase in GDP per capita in this regard.

A nearly perfect positive correlation was observed between the WEEE accumulation indicator per capita and the mass of EEE introduced in the previous year on a national scale, despite a decrease in population. Matsumoto (2011) showed another correlation between an increase in the waste collection rate and population. A statistically significant relationship was also found between the WEEE accumulation indicator and the annual disposable income per capita, with a very high determination indicator. In this context, Kusch and Hills (2017) identified a linear relationship between WEEE generation and GDP. According to Azizi et al. (2023), the noted indicators can improve the current e-waste management system.

Substantial increases in the accumulation of WEEE per capita and GDP indicate that its recovery and recycling are crucial in the development of the circular economy (Zhang and Zhang 2018), which should foster economic gains from savings by reducing energy and material costs (Wang et al. 2019). De Meester et al. (2019) showed that WEEE is a key resource in the circular economy due to factors such as the content of valuable materials such as metals. Using the example of Brazil, Fernandes et al. (2021) showed a significantly different solution for WEEE management: storage. In turn, using the example of Germany, Diedler et al. (2018) considered a good example of good practice in the way that EEE is imported/sold and WEEE collected, which benefits interested parties such as logistics service providers or recycling plants. However, Sousa et al. (2018) showed that in European countries, the collection, treatment, and recycling of WEEE are being included in the promotion of best practices to increase the efficiency of WEEE management systems and achieve European standards and targets for collection and treatment, including recovery and recycling rates and in Poland, the promotion of WEEE repair and reuse, and the proper collection of waste equipment (KPGO 2028).

## Conclusion

Based on the analysis of the WEEE economy in Poland during the 2014–2018 period, a 16.64% increase in the mass of collected equipment indicates the need to develop recycling technology. Although the largest annual mass of WEEE collected was 283,399 Mg, the amount of EEE introduced to the market was higher, as confirmed by the total mass of 607,240 Mg, as well as the average exceeding 500,000 Mg. The largest share in the mass of WEEE collected (235,508.52 Mg) was for waste code 20 01 36 (i.e. WEEE other than those listed in 20 01 21*, 20 01 23*, and 20 01 35*). On the national scale, the differentiation of WEEE accumulation in individual voivodeships is noticeable, as indicated by the highest value of the annual waste weight accumulation indicator of 45.98 kg per capita in one of the smallest voivodeships in Poland, with the minimum collection target of 4 kg per person per year in the EU, as set out in Directive 2002/96/EC (EC 2003). The highest results were generally found in the last analysed year, which confirms an increasing trend overall. In the analysed period, WEEE in the country was typically collected at points for the selective collection of municipal waste and shops selling electrical and electronic devices, from where it was sent to recovery and recycling installations. This process was funded by EEE producers. The rise in generated e-waste, and, more precisely, the increase in the mass of WEEE collected per unit GDP by 208 Mg billion USD^−1^ and the mass of collected WEEE per disposable income increasing by 4.7 Mg PLN^−1^, affected the cost of WEEE management in Poland. The increase in annual GDP and annual DIC significantly influences the mass of introduced EEE and collected WEEE per capita, despite a noticeable decrease in population. These results confirm that monitoring individual indicators of waste accumulation, including socioeconomic factors, can help to identify the main challenges in the development of WEEE recovery and recycling for the transition to a circular economy. A global synergy of activities amongst all players is required, from manufacturers to consumers, to properly tackle the WEEE management challenge. This is essential because collaboration between all players and policy frameworks is required to solve the WEEE challenge (Shittu et al. 2021).

## Data Availability

The datasets used and/or analysed during the current study are available from the corresponding author on reasonable request.
